# High‐pressure leakage of pleural fluid through the healed entry site of the indwelling pleural catheter from undrained locules

**DOI:** 10.1002/rcr2.587

**Published:** 2020-05-17

**Authors:** Ka Pang Chan, Ka Ching Joyce Ng, Chi To Kevin Li

**Affiliations:** ^1^ Department of Medicine & Therapeutics Chinese University of Hong Kong Hong Kong; ^2^ Department of Medicine & Therapeutics Prince of Wales Hospital Hong Kong; ^3^ Department of Medicine & Geriatrics Sha Tin Hospital Hong Kong

**Keywords:** Fluid leakage, indwelling pleural catheter, loculated pleural effusion, malignant pleural effusion

## Abstract

The indwelling pleural catheter (IPC) is an established treatment for recurrent pleural effusion. Fluid leakage through the IPC insertion tract has been reported, but its occurrence is only limited to a short period after the procedure. Besides, the drainage efficacy of IPC may be limited by the presence of loculation in the pleural space, especially when intrapleural fibrinolytic is contraindicated. We report a case of fluid leakage through the healed entry site of IPC due to high pressure built from undrained pleural fluid locules, which was successfully treated with an additional drain targeting the largest undrained locule.

## Graphical abstract

This is the first case report for fluid leakage through a healed entry site of the indwelling pleural catheter due to high pressure built from undrained pleural fluid locules. The condition was successfully treated with an additional drain targeting the largest undrained locule.

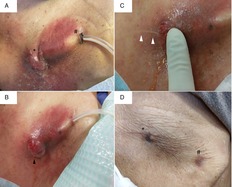

## Introduction

Malignant pleural effusion (MPE) is a common condition in metastatic malignancy and necessitates repeated pleural drainages. The indwelling pleural catheter (IPC) is an established treatment for MPE to minimize the need for repeated pleural intervention. However, its drainage effectiveness can be limited by fluid loculation [1], and various complications may happen after placement, including fluid leakage, wound infection, and catheter tract metastasis (CTM) [2, 3, 4]. We report a case of fluid leakage through the healed entry site of IPC due to high intrapleural pressure built inside an undrained pleural fluid locule, which mimics wound infection and CTM.

## Case Report

Our patient, an 88‐year‐old gentleman, presented to the Department of Oncology for recurrent right‐sided MPE due to metastatic lung adenocarcinoma. This was his third episode of recurrent symptomatic MPE in the past two months and all prior episodes were treated by simple pleural drainages only. The pleural fluid became more loculated on the chest X‐ray (CXR) after repeated procedures over these two months.

**Figure 1:**
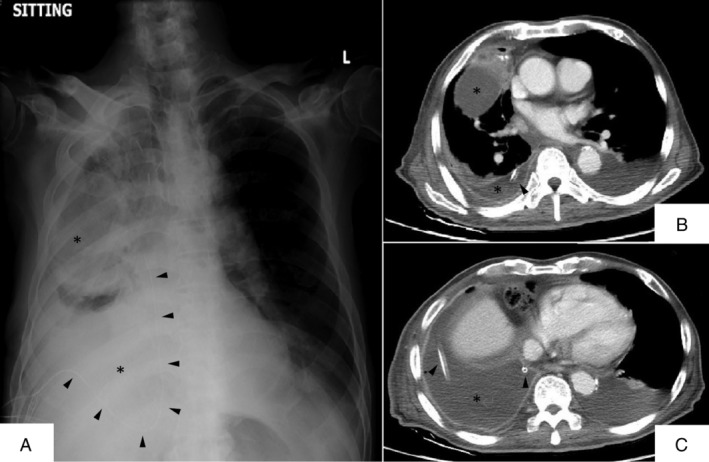
Chest X‐ray (A) and computed tomography images (B and C) showing the indwelling pleural catheter (arrowheads) and loculated right pleural effusion (*).

The Respiratory Division was consulted for the placement of IPC. Pre‐insertion thoracic ultrasound (TUS) revealed a heavily septated right pleural space containing multiple fluid locules. The patient consented for the placement of IPC and acknowledged the possibility of incomplete pleural fluid drainage due to septated pleural space. IPC was inserted successfully to the largest locule at the laterobasal pleural space and allowed smooth fluid drainage. However, the right hemithorax remained partially opacified on imaging due to non‐drainable fluid locules (Fig. 1). A trial of intrapleural urokinase was injected through the IPC to break the septation, but it was not further attempted due to increased haemorrhagic appearance of pleural fluid output and lack of radiological improvement. As the patient experienced symptomatic relief from IPC drainage, it was decided not to proceed for further pleural intervention unless deterioration of respiratory symptoms occurs. The daily IPC output was 500–1000 mL and IPC wounds healed afterwards.

**Figure 2:**
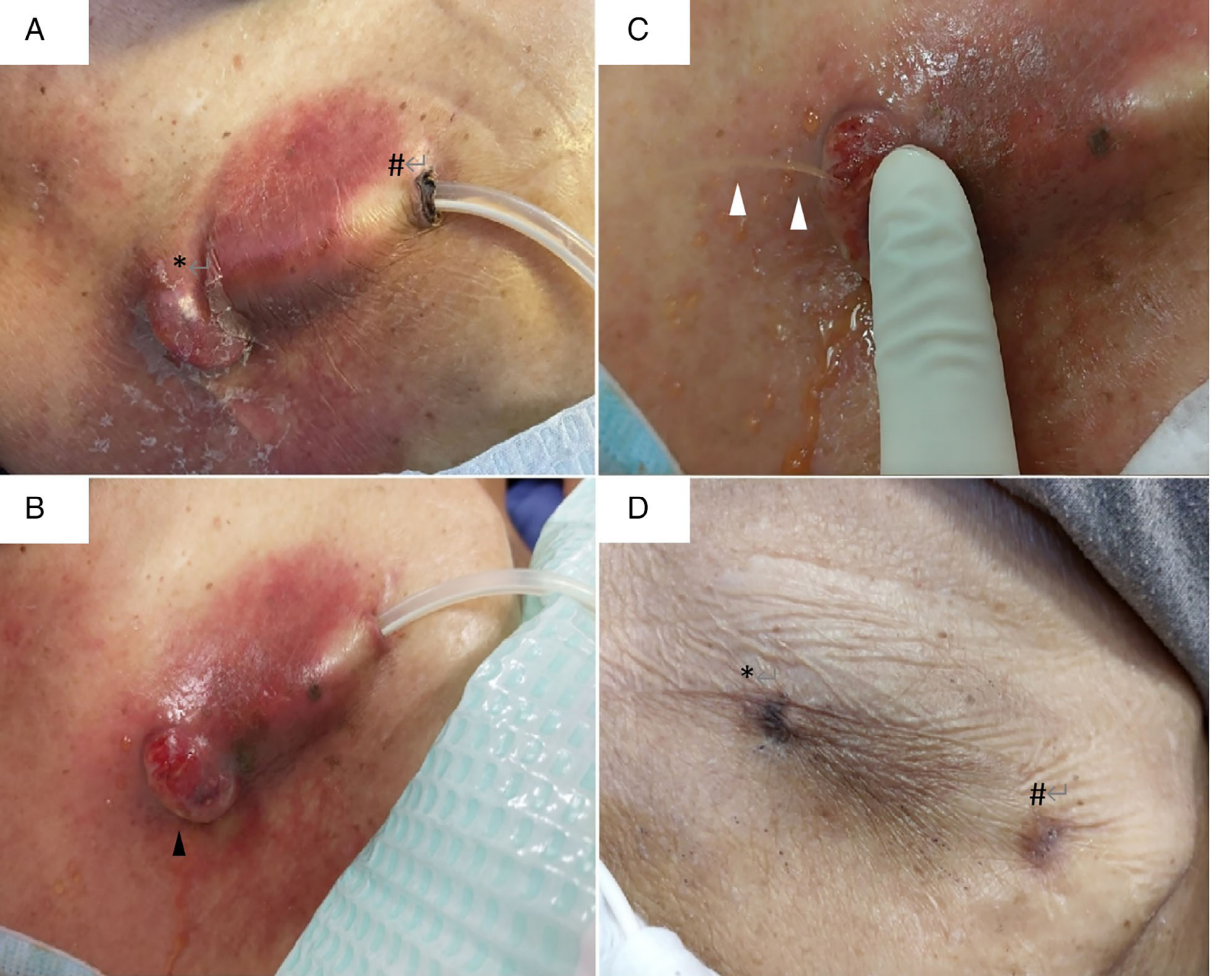
Clinical appearances of the entry and exit sites of the indwelling pleural catheter (IPC). (A) An area of erythema and subcutaneous oedema at the entry site (*) and along the subcutaneous tract of IPC, sparing the exit site (#). The skin of the entry site remains intact. (B) Further increase in subcutaneous oedema and an eruption of a bulla (black arrowhead) at the entry site. (C) Spurting of fluid (white arrowheads) due to high pressure built inside the bulla, together with ongoing oozing from it. (D) Both the entry (*) and exit (#) sites of IPC healed after catheter removal.

At the eighth week, there was a reduction of output to less than a 50 mL per day, together with pain at the healed IPC entry site and progressive increase in shortness of breath. Examination revealed an area of erythematous oedema at the healed entry site of IPC, while sparing the exit site (Fig. 2A). There were no signs of pus collection, granuloma formation, or neoplastic deposits. A course of co‐amoxiclav was given but the area of erythema continued to enlarge, with an eruption of bulla two days later (Fig. 2B). The bulla finally ruptured with continuous serous fluid discharge and intermittent spurting of fluid (Fig. 2C) (Video S1). The IPC was confirmed patent by saline flushing but only minimal fluid could be aspirated. The right lung field was completely opacified on CXR and TUS demonstrated multiple fluid locules near the entry site which may not be drained by the IPC. A new chest drain was inserted targeting the largest locule at the posterolateral pleural space, and achieved symptomatic and radiological improvement. The fluid oozing from the entry site became minimal over the next few days. It was then decided to remove IPC as there was no more output after new drain insertion and to promote healing of the entry site. Dense fibrosis around the catheter polyester cuff was noted during the catheter removal procedure and the catheter was freed after dissection of the subcutaneous tract. The exit site was stitched and entry site was covered by a stoma bag. The erythematous oedema at the entry site and the fluid oozing both resolved slowly. The patient was then discharged with the newly inserted pleural drain.

The patient returned to the clinic four weeks later and demonstrated complete healing of the IPC entry and exit sites (Fig. 2D). The newly inserted pleural drain was draining 500–1000 mL pleural fluid each day without signs of pleural fluid leakage.

## Discussion

Persistent fluid leakage through the IPC entry site shortly after insertion has been reported [3, 4]. It is due to the high intrapleural pressure from the undrained pleural effusion, which can be avoided by drainage prior to and immediately after the insertion procedure [3]. To the best of our knowledge, leakage along the IPC insertion tract and rupture through the healed entry site has never been reported. The leakage is unlikely to happen if the IPC functions well and drains free‐flowing pleural effusion. In our patient, the undrained pleural fluid locule slowly built up the pressure but could not escape through the fibrosed cuff to the exit site. The fluid tracked around the catheter insertion site and ruptured through the skin. The use of fibrinolytic may help to break the locule and enhance the drainage, but it was not further attempted due to high bleeding risk in this case. An additional drain was therefore inserted to relieve the intrapleural pressure, which indirectly stopped the leakage.

The occurrence of erythematous induration around the entry site should raise the suspicion of CTM and wound infection. However, the rapid onset of symptoms argued against the possibility of CTM, while the delayed occurrence at a healed wound and a failed response to antibiotic also excluded the diagnosis of wound infection. Combining the prior drainage history and radiological findings, this rare complication of leakage through a healed tract should be considered and an additional drain can relieve both the symptoms and leakage.

### Disclosure Statement

Appropriate written informed consent was obtained for publication of this case report and accompanying images.

## Supporting information


**Video S1.** Spurting of fluid from a bulla at the entry site of indwelling pleural catheter (IPC). The bulla situated at the entry site of IPC with a surrounding area of erythema and subcutaneous oedema. Spurting and continuous oozing of fluid from the bulla due to high pressure built inside it.
